# Supplementary figures and images for: Comparative evaluation of machine learning algorithms for phishing site detection
Source: PeerJ Comput Sci. 2024 Jun 24;10:e2131. doi: 10.7717/peerj-cs.2131 (PMC11232597; doi:10.7717/peerj-cs.2131)

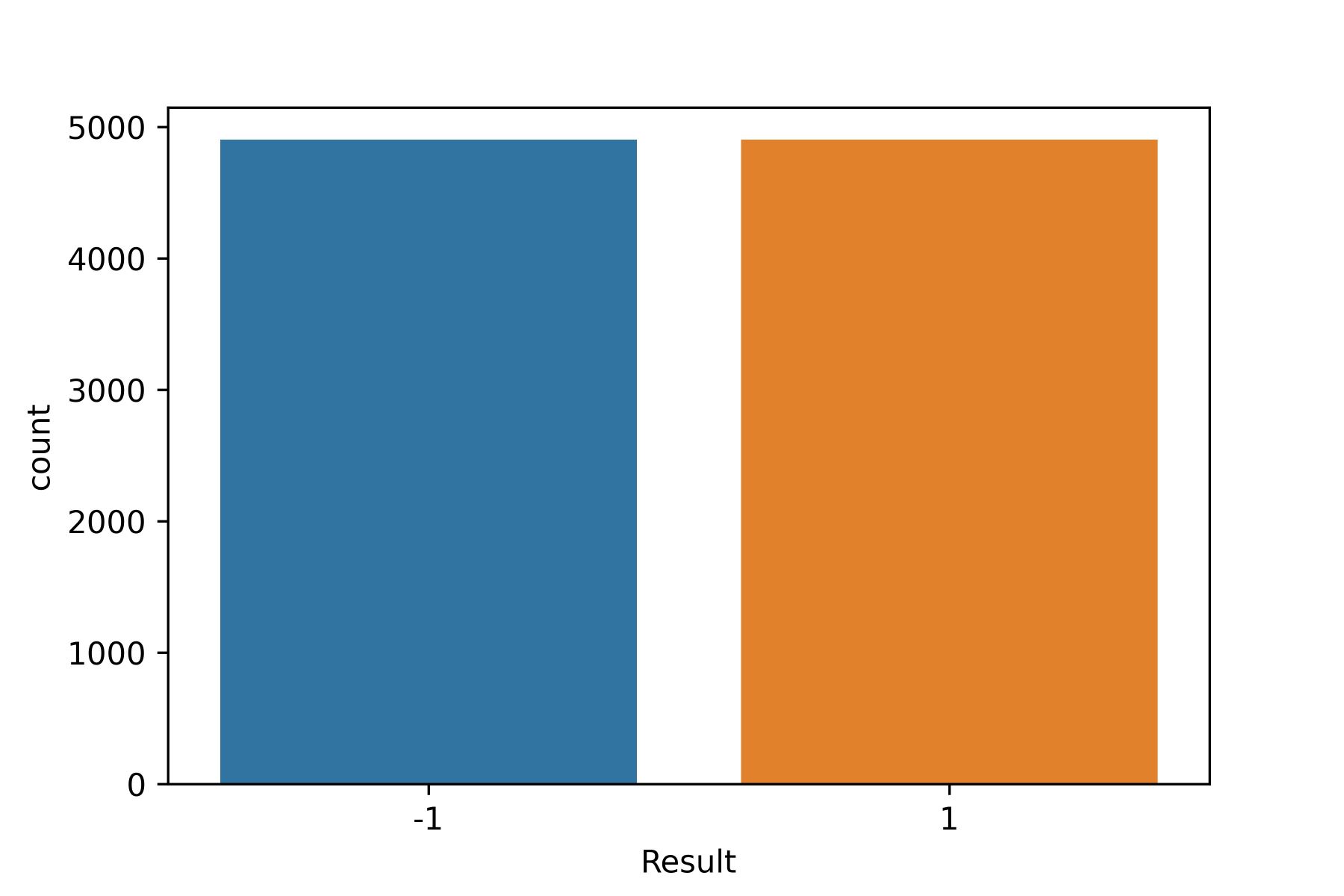

Supplement: Figure S1 [file peerj-cs-10-2131-s001.jpg]

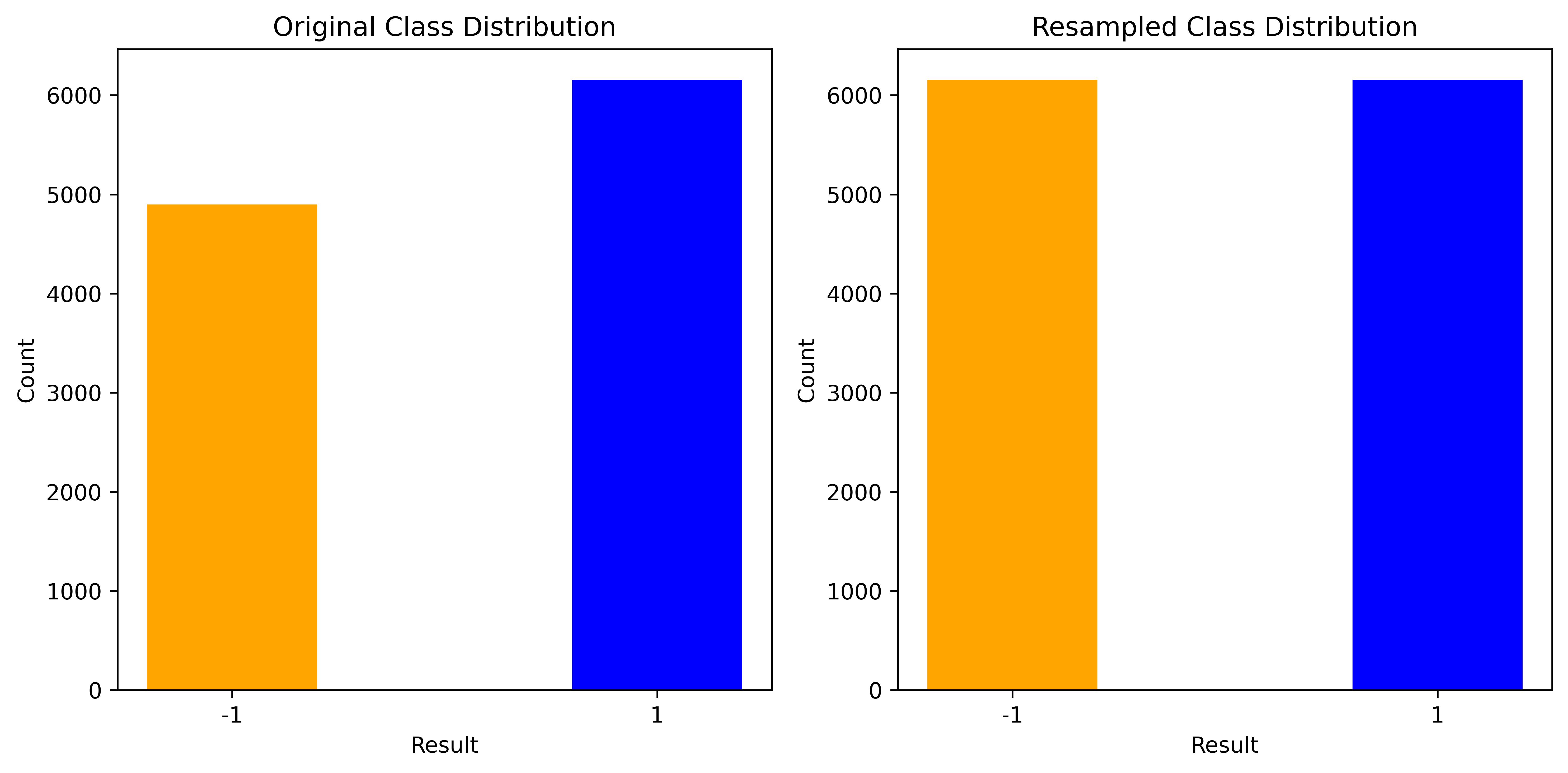

Supplement: Figure S2 [file peerj-cs-10-2131-s002.jpeg]

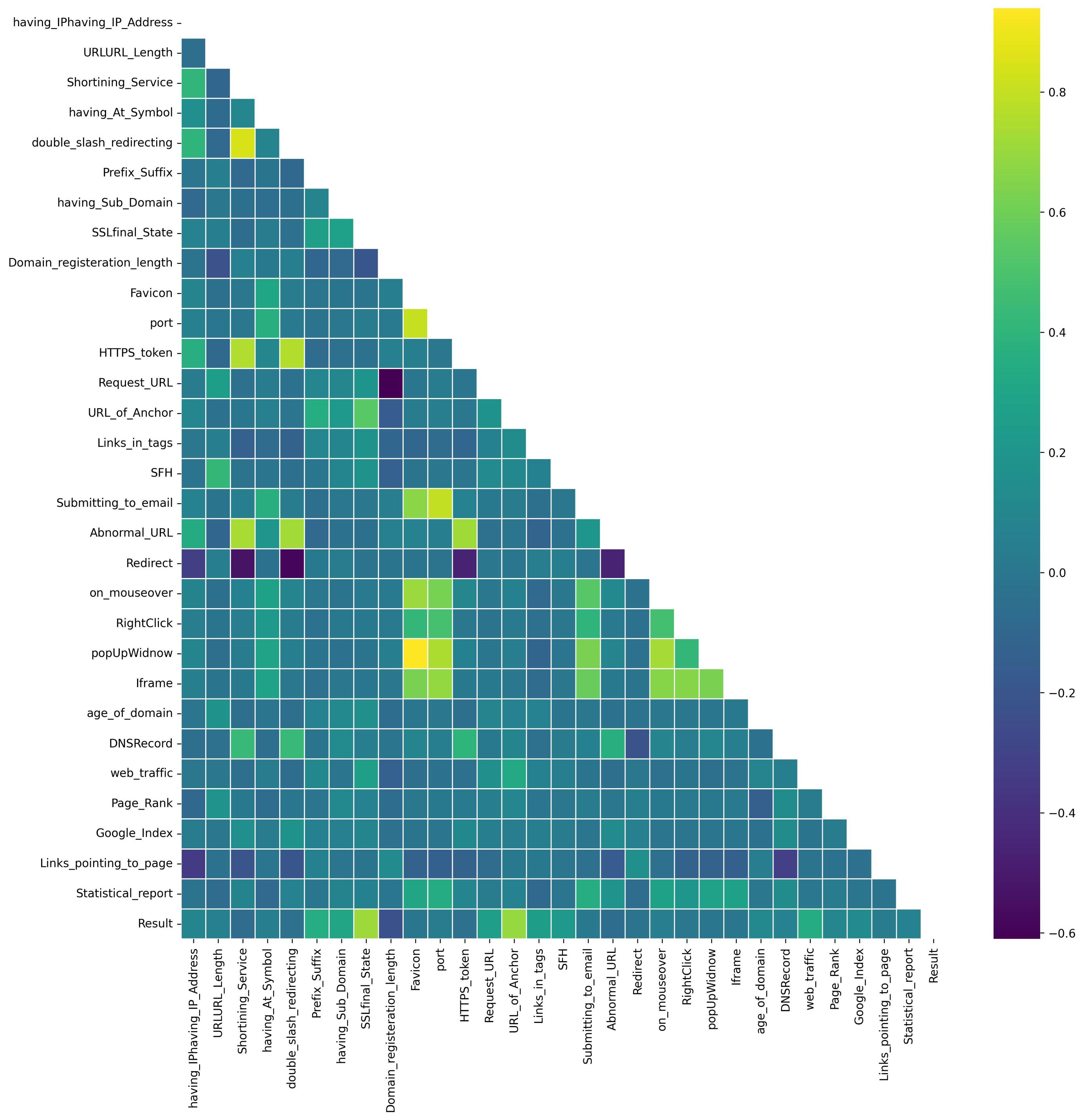

Supplement: Figure S3 [file peerj-cs-10-2131-s003.jpg]

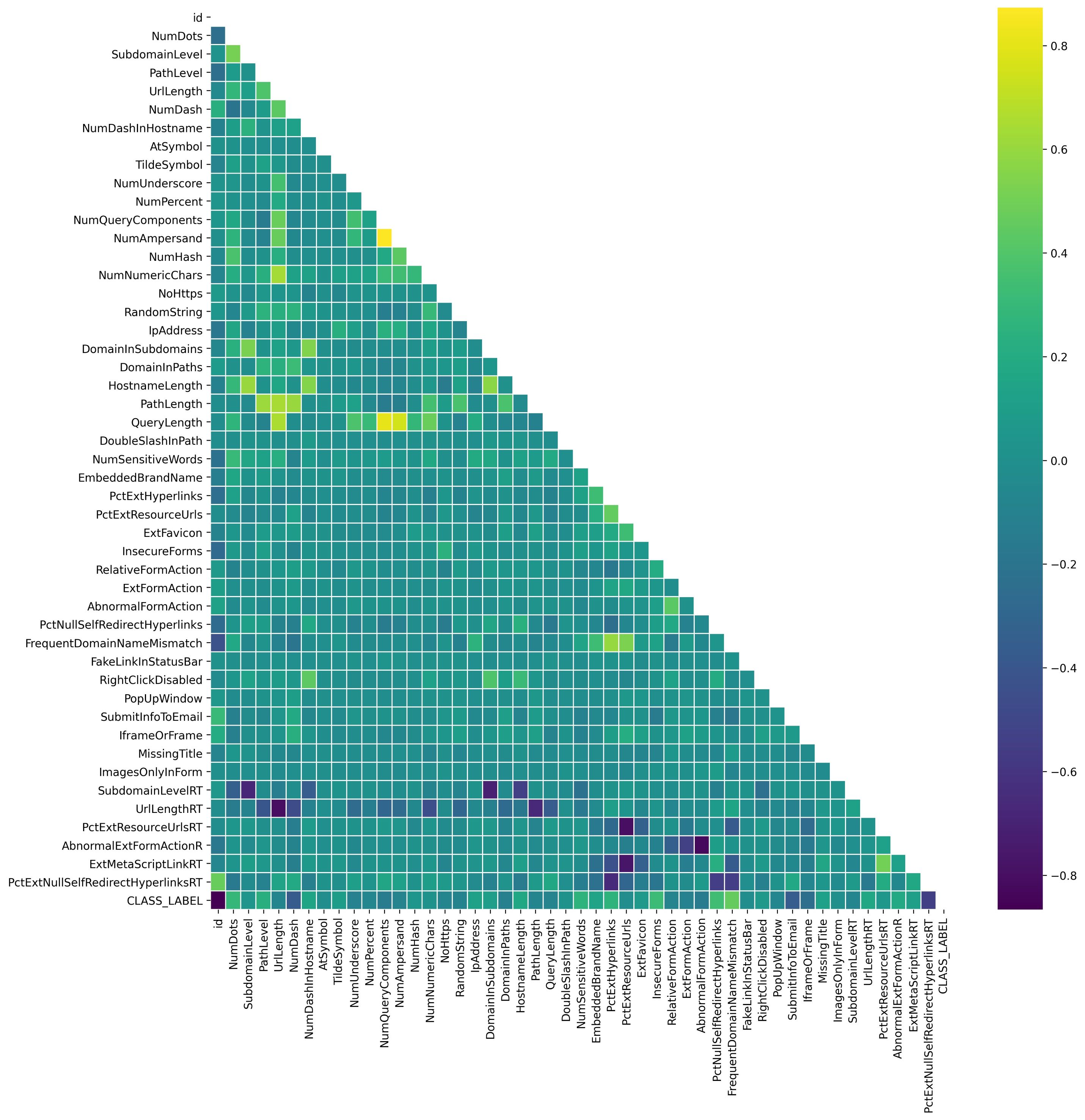

Supplement: Figure S4 [file peerj-cs-10-2131-s004.jpg]

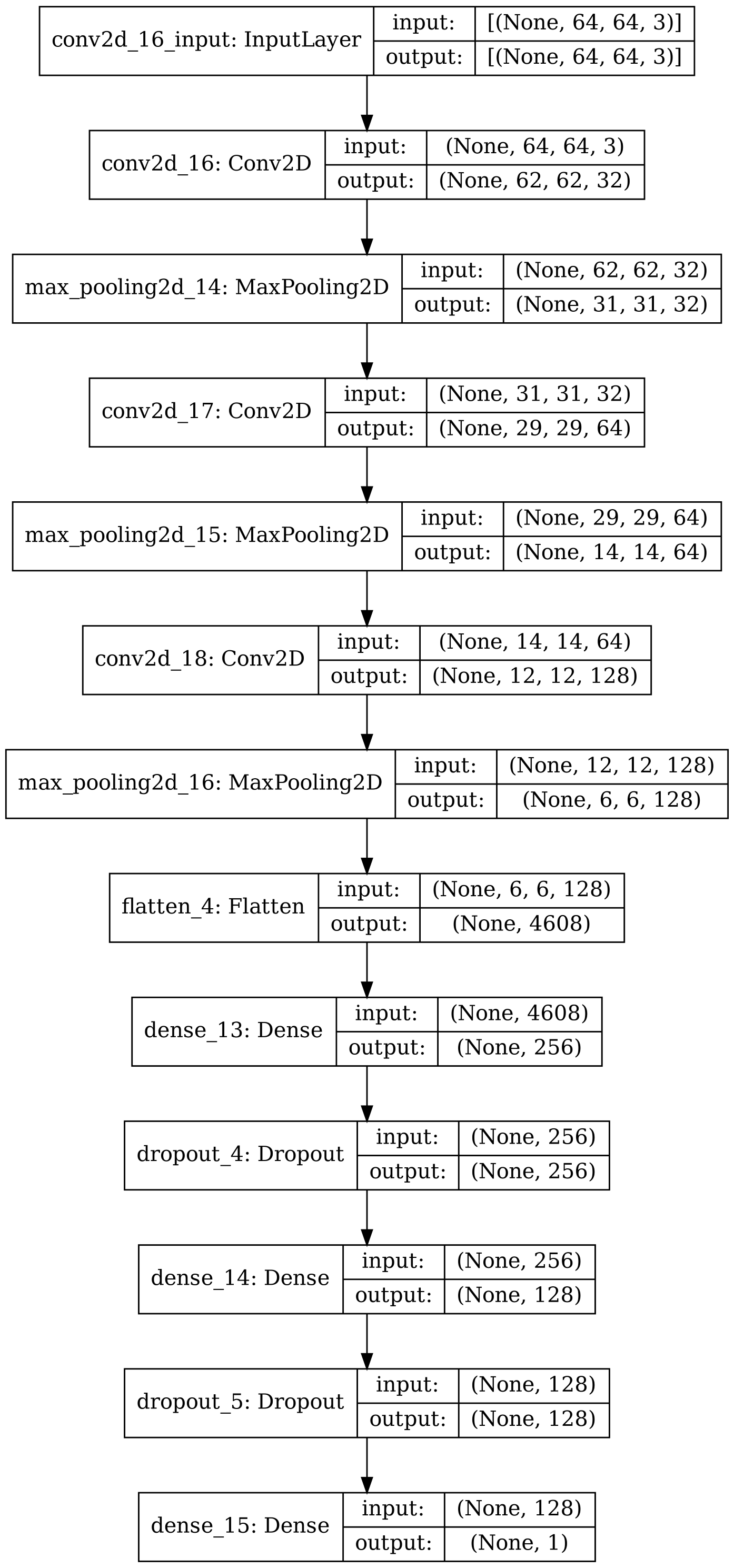

Supplement: Figure S5 [file peerj-cs-10-2131-s005.png]

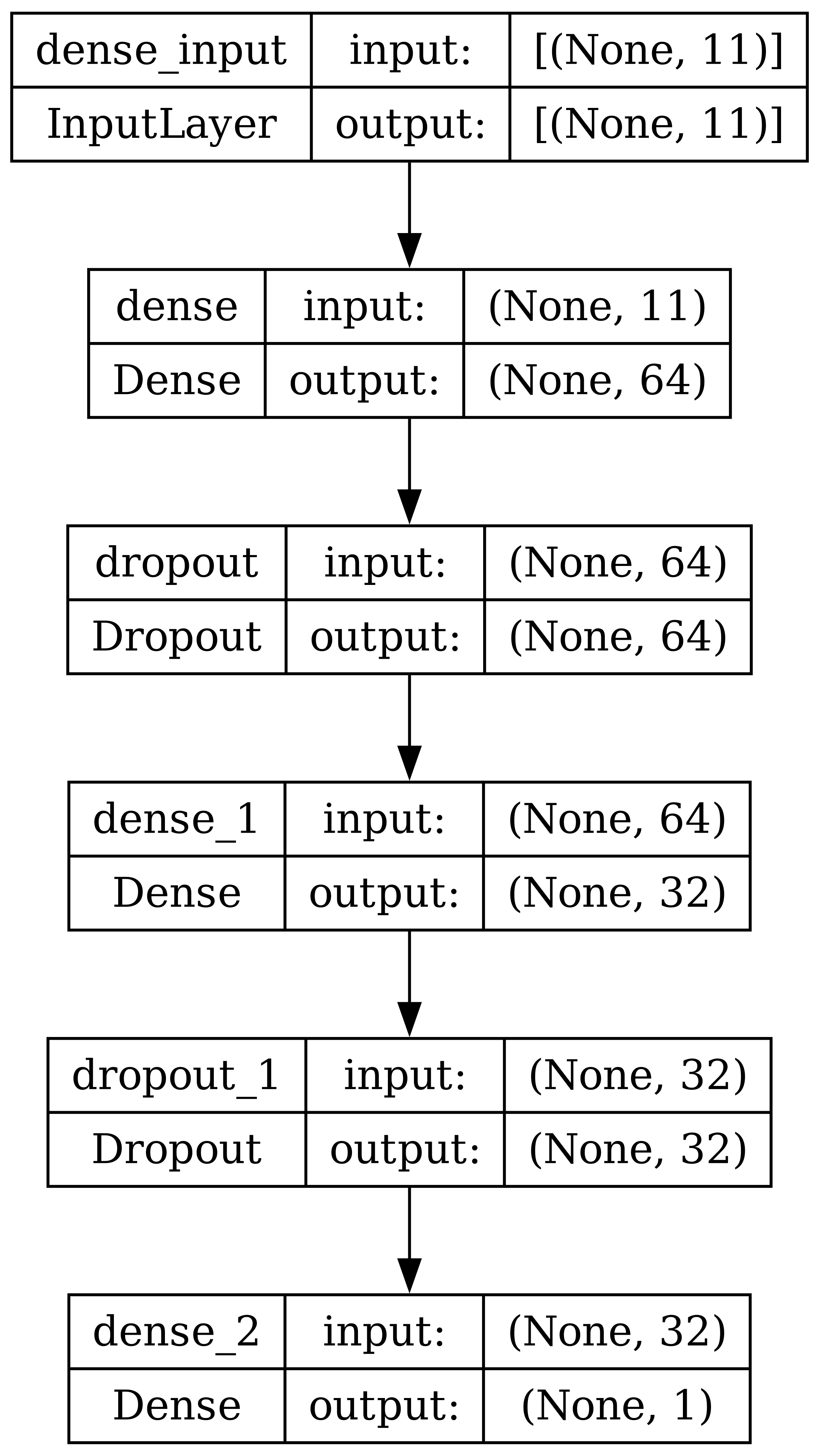

Supplement: Figure S6 [file peerj-cs-10-2131-s006.png]

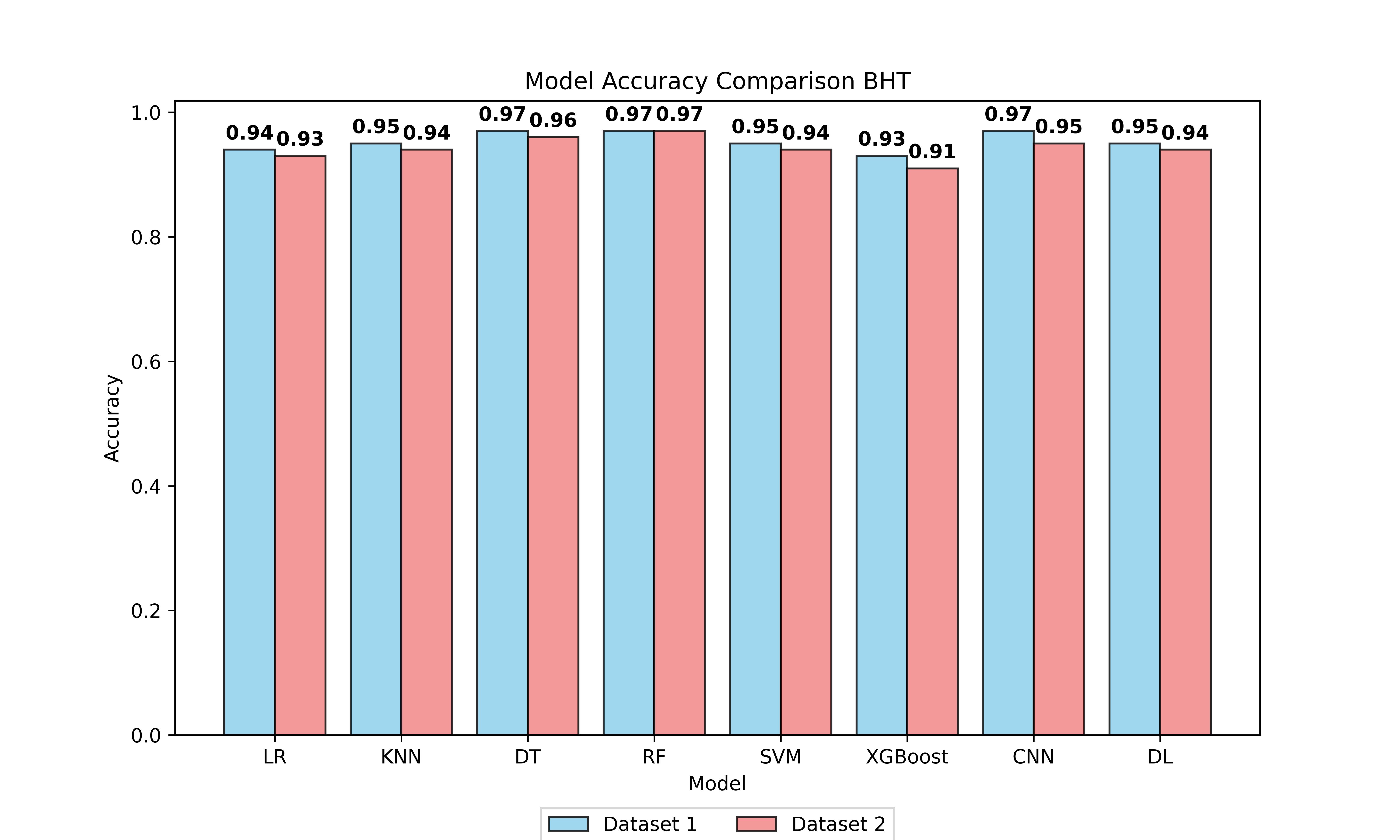

Supplement: Figure S7 [file peerj-cs-10-2131-s007.jpeg]
