# Supplementary material for: Comparative evaluation of machine learning algorithms for phishing site detection
Source: PeerJ Comput Sci. 2024 Jun 24;10:e2131. doi: 10.7717/peerj-cs.2131 (PMC11232597; doi:10.7717/peerj-cs.2131)
Supplement: Table S1 [file peerj-cs-10-2131-s008.docx]

**Table S1.** Details of the literature review.

| **Studies** | **Contributions** | **Accuracy** | **Limitations** |
| --- | --- | --- | --- |
| Hung Le et al [5] | Presented a DL-based URL detector that can provide insights from URLs. | For Phishtank, the accuracy was 93.8%, while for the crawler, the accuracy was 93.6%. | Deep learning techniques require more time to complete tasks. Furthermore, the system evaluates the URL and compares it to the library to generate an output. |
| Hassan Y.A.[7] | Proposed a URL detector for classifying websites and identifying phishing sites. They utilized the GA method to improve performance. | The accuracy ranged from 95.62% up to 98.07%. | Despite its better performance, the URL detector based on the genetic algorithm (GA) had excessively long prediction times for complex sets of URLs. |
| Kumar J. et al [8] | Presented a URL detector based on a blacklisted URL and successfully differentiated between legitimate and malicious websites using a lexical feature approach. | The Bayes classifier had an accuracy of 98%. | The effectiveness of the detector with residing URLs may be affected by the use of an older dataset by the authors. |
| Rao RS. et al [9] | The page attributes they considered included logos, favicons, texts, and styles. | Jail-Phish achieved an accuracy of 98.61%, a TPR of 97.77%, and an FPR of 0.64%, according to the experimental results. | The method used a web server to update page characteristics, which adversely affected the performance of the detection system. |
| Aljofey A. et al [10] | Proposed a CNN-based system for detecting phishing pages and used a sequential pattern to locate URLs. | Obtained accuracies of 98.58%, 95.46%, and 95.22%, respectively. | According to the research, CNN outperforms text in terms of image retrieval. |
| Suleiman Y et al [17]. | Used a CNN-based model to detect phishing sites using a dataset containing 11055 records. | Accuracy was 97.1% with an F1-score of 0.974. | The training process cannot achieve high results simply by automating the search and parameter selection, such as length and the number of filters. |
| Mohammad et al [18] | Employed the 'hold-out' validation approach and used the 'log sigmoid' activation function. | The training set accuracy was 94.07%, the validation set accuracy was 91.31%, and the testing set accuracy was 92.18%. | The model needs continuous updating, and feature importance was absent. |
| Faisal Khan et al [19] | Utilized minimal features, including feature extraction and classification techniques, and used accuracy and error rates as computational metrics. | The training accuracy was 99.71%, and the test accuracy was 99.13%. | The system relies on third-party capabilities, and if these capabilities are not available, the system may fail. Consequently, the validation of the system may be inaccurately analyzed. Additionally, if malicious phishing sites use embedded objects to replace text, the model may fail to detect them. |
| Dunlop et al [20] | Introduced the concept of using optical character recognition to convert logos and screenshots of images into text, thereby reducing the two-query approach to a single query. | The total accuracy was 98%, and the overall detection accuracy was 100%. | Converting images to text incurs delays in rendering web pages and requires both time and money. Furthermore, the client-side OCR processing makes it platform-dependent. |
| Varshney et al [21] | Used URL and page title information to develop a useful search term for recognizing phishing websites. They improved and compared search results and created a functional prototype (LPD) for Google Chrome. | TPR was 99.5%, and the TNR was 100%. The accuracy was 92.4%. | Due to its lightweight nature and reliance on only two features, the LPD may generate false positives for benign recently launched websites. The authors suggest including additional features in future work while maintaining resource effectiveness. |
| Jain A. et al [22] | Employed both SVM and NB algorithms to determine malicious websites. | For the set of 15,000 training URLs, the accuracy was 76.04%. For the set of 25,000 URLs, the accuracy was 91.28%. | NB and SVM are both slow learners that do not retain previous outcomes in memory. As a result, the effectiveness of this URL detector might be diminished. |
| Luong Anh et.al [23] | Identified six minimal features and aimed to achieve a high degree of accuracy. | Phishtank, with 11,660 phishing sites, had an accuracy of 97.16%. | The operation of this real-time client-side tool is challenging due to its use of various heuristics. Additionally, it heavily relies on third parties. |
| Ramesh et al. [24] | Employed DNS lookups and hyperlinks from HTML resources to identify the target domain set for matching | Phishtank, the accuracy was 99.62%. | Finding associations, both direct and indirect, for each webpage a user visits take time and effort. Furthermore, the process is complex and system-dependent due to the DNS requirement. |
| Singh et al. [25] | Tested Madaline and backpropagation for phishing website classification by training neural networks on top of SVM using more than 15 features. Claimed that Adaline was more efficient and precise at classification. | For the 179 legitimate websites from Alexa, the accuracy was 99.14%. | Using SVM and neural networks for multi-stage classification requires additional resources. |
