# Supplementary material for: Comparative evaluation of machine learning algorithms for phishing site detection
Source: PeerJ Comput Sci. 2024 Jun 24;10:e2131. doi: 10.7717/peerj-cs.2131 (PMC11232597; doi:10.7717/peerj-cs.2131)
Supplement: Table S2 [file peerj-cs-10-2131-s009.docx]

**Table S2.** Address bar-based features for dataset 2

| Features of Address Bar | Description | Remarks |
| --- | --- | --- |
| **IP address** | In the URL, the IP address is displayed instead of the domain name. | http://192.112.68.17/Clickme.html |
| **Long URL to hide the suspicious part** | Long URLs were used to conceal suspicious parts. | http://topmoves.com.br/3f/aze/ab51e2e319e51502f416dbe46b773a5e/?cmd=_home&dispatch=11004d58f5b74f8dc1e7c2e8dd4105e811004d58f5b74f8dc1e7c2e8dd4105e8@fake.website.html |
| URL shortening | URLs can be significantly shortened using services like TinyURL. | bit.ly/81GDXSv9 |
| URL’s with @ symbol | The @ symbol indicates that the previous text in the URL should be ignored, and the actual text starts after the @ symbol. | https://www.cbd.ae/personal/bank@redirect=fake.html |
| **// Redirecting** | The use of "//" in the URL is for redirecting to another website. | “http://www.good.com//http://www.bad.com |
| Dash symbol - | Phishing URLs often contain hyphens (-) in the domain name to make users believe they are using a legitimate website. | https://www.al-rajhibank.com.sa |
| Using sub-domains and multi-subdomains | A higher number of dots in the URL can indicate multiple domains, which may be associated with phishing. | https://www.ku.ac.ae/ /students/ |
| HTTPS | Having only HTTPS in the URL does not guarantee the legitimacy of the website. | Generally, 2 years of expiry for reputed certificates. |
| Length of domain registration | Phishing websites have a short lifespan. | Legitimate domains were only utilized for one year. |
| Favicon | A favicon is an icon or graphic image displayed on a webpage. | Favicon can be loaded from an illegitimate domain in a URL for phishing |
| **Non-Standard Port** | NAT and firewalls block major ports and only open the required ones. | Opening all ports can be an open invitation for the phishers to run almost any service to threaten the system. |
| Illusionary HTTPS | Phishers can use symbols like "-" and "etc" to mimic HTTPS URLs. | http://https-www-alrajhi-com-sendlogindetails.php |
