# Supplementary material for: Comparative evaluation of machine learning algorithms for phishing site detection
Source: PeerJ Comput Sci. 2024 Jun 24;10:e2131. doi: 10.7717/peerj-cs.2131 (PMC11232597; doi:10.7717/peerj-cs.2131)
Supplement: Table S3 [file peerj-cs-10-2131-s010.docx]

| Address Bar | Description | Remarks |
| --- | --- | --- |
| **Request URL** | It is defined by the <r> tag and functions similarly to the request URL. | These objects are within the webpage and have the same domain, whereas, in phishing, they request objects from other domains. |
| Anchor’s URL | It is defined by the <a> tag and functions similarly to the request URL. | The domain names may be different for the <a> tags and the website for phishing. |
| Links in <Meta>, <Script> and <Link> tags | It is common for regular web pages to utilize these tags. However, these tags may redirect to illegitimate web pages. | Link=www.fake.com |
| **Server Form Handler (SFH)** | If it is blank or contains a different domain name, it may be a phishing attempt. | The empty () method can be used with the get or post method to prevent it. |
| Information submission through email | Phishers retrieve information through email IDs using functions like mail() or mailto(). | URLs that contain functions like mail() or mailto() can be categorized as phishing attempts. |
| Abnormal URL | The URL does not include the hostname. | Prevention can be achieved by using the Whois dataset. |

**Table S3.** Abnormal features for the dataset 2
