# Supplementary material for: Comparative evaluation of machine learning algorithms for phishing site detection
Source: PeerJ Comput Sci. 2024 Jun 24;10:e2131. doi: 10.7717/peerj-cs.2131 (PMC11232597; doi:10.7717/peerj-cs.2131)
Supplement: Table S4 [file peerj-cs-10-2131-s011.docx]

**Table S4.** Phishing feature and label information for dataset 1.

| # | Column | Non-Null | Datatype | # | Column | Non-Null | Datatype |
| --- | --- | --- | --- | --- | --- | --- | --- |
| 0 | index | 11055 | int64 | 16 | SFH | 11055 | int64 |
| 1 | having_IPhaving_IP_Address | 11055 | int64 | 17 | Submitting_to_email | 11055 | int64 |
| 2 | URLURL_Length | 11055 | int64 | 18 | Abnormal_URL | 11055 | int64 |
| 3 | Shortining_Service | 11055 | int64 | 19 | Redirect | 11055 | int64 |
| 4 | having_At_Symbol | 11055 | int64 | 20 | on_mouseover | 11055 | int64 |
| 5 | double_slash_redirecting | 11055 | int64 | 21 | RightClick | 11055 | int64 |
| 6 | Prefix_Suffix | 11055 | int64 | 22 | popupWindow | 11055 | int64 |
| 7 | having_Sub_Domain | 11055 | int64 | 23 | Iframe | 11055 | int64 |
| 8 | SSLfinal_State | 11055 | int64 | 24 | age_of_domain | 11055 | int64 |
| 9 | Domain_registeration_length | 11055 | int64 | 25 | DNSRecord | 11055 | int64 |
| 10 | Favicon | 11055 | int64 | 26 | web_traffic | 11055 | int64 |
| 11 | port | 11055 | int64 | 27 | Page_Rank | 11055 | int64 |
| 12 | HTTPS_token | 11055 | int64 | 28 | Google_Index | 11055 | int64 |
| 13 | Request_URL | 11055 | int64 | 29 | Links_pointing_to_page | 11055 | int64 |
| 14 | URL_of_Anchor | 11055 | int64 | 30 | Statistical_report | 11055 | int64 |
| 15 | Links_in_tags | 11055 | int64 | 31 | Result | 11055 | int64 |
