# Supplementary material for: Comparative evaluation of machine learning algorithms for phishing site detection
Source: PeerJ Comput Sci. 2024 Jun 24;10:e2131. doi: 10.7717/peerj-cs.2131 (PMC11232597; doi:10.7717/peerj-cs.2131)
Supplement: Table S5 [file peerj-cs-10-2131-s012.docx]

**Table S5.** Phishing feature and label information for dataset 2

| # | Column | Non-Null | Datatype | # | Column | Non-Null | Datatype |
| --- | --- | --- | --- | --- | --- | --- | --- |
| 0 | id | 10000 | int64 | 25 | NumSensitiveWords | 10000 | int64 |
| 1 | NumDots | 10000 | int64 | 26 | EmbeddedBrandName | 10000 | int64 |
| 2 | SubdomainLevel | 10000 | int64 | 27 | PctExtHyperlinks | 10000 | float64 |
| 3 | PathLevel | 10000 | int64 | 28 | PctExtResourceUrls | 10000 | float64 |
| 4 | UrlLength | 10000 | int64 | 29 | ExtFavicon | 10000 | int64 |
| 5 | NumDash | 10000 | int64 | 30 | InsecureForms | 10000 | int64 |
| 6 | NumDashInHostname | 10000 | int64 | 31 | RelativeFormAction | 10000 | int64 |
| 7 | AtSymbol | 10000 | int64 | 32 | ExtFormAction | 10000 | int64 |
| 8 | TildeSymbol | 10000 | int64 | 33 | AbnormalFormAction | 10000 | int64 |
| 9 | NumUnderscore | 10000 | int64 | 34 | PctNullSelfRedirectHyperlinks | 10000 | float64 |
| 10 | NumPercent | 10000 | int64 | 35 | FrequentDomainNameMismatch | 10000 | int64 |
| 11 | NumQueryComponents | 10000 | int64 | 36 | FakeLinkInStatusBar | 10000 | int64 |
| 12 | NumAmpersand | 10000 | int64 | 37 | RightClickDisabled | 10000 | int64 |
| 13 | NumHash | 10000 | int64 | 38 | PopUpWindow | 10000 | int64 |
| 14 | NumNumericChars | 10000 | int64 | 39 | SubmitInfoToEmail | 10000 | int64 |
| 15 | NoHttps | 10000 | int64 | 40 | IframeOrFrame | 10000 | int64 |
| 16 | RandomString | 10000 | int64 | 41 | MissingTitle | 10000 | int64 |
| 17 | IpAddress | 10000 | int64 | 42 | ImagesOnlyInForm | 10000 | int64 |
| 18 | DomainInSubdomains | 10000 | int64 | 43 | SubdomainLevelRT | 10000 | int64 |
| 19 | DomainInPaths | 10000 | int64 | 44 | UrlLengthRT | 10000 | int64 |
| 20 | HttpsInHostname | 10000 | int64 | 45 | PctExtResourceUrlsRT | 10000 | int64 |
| 21 | HostnameLength | 10000 | int64 | 46 | AbnormalExtFormActionR | 10000 | int64 |
| 22 | PathLength | 10000 | int64 | 47 | ExtMetaScriptLinkRT | 10000 | int64 |
| 23 | QueryLength | 10000 | int64 | 48 | PctExtNullSelfRedirectHyperlinksRT | 10000 | int64 |
| 24 | DoubleSlashInPath | 10000 | int64 | 49 | CLASS_LABEL | 10000 | int64 |
