# Supplementary material for: Comparative evaluation of machine learning algorithms for phishing site detection
Source: PeerJ Comput Sci. 2024 Jun 24;10:e2131. doi: 10.7717/peerj-cs.2131 (PMC11232597; doi:10.7717/peerj-cs.2131)
Supplement: Table S6 [file peerj-cs-10-2131-s013.docx]

**Table S6.** Feature for both datasets with the highest scores based on TU and PBCC

| Dataset 1 | | | Dataset 2 | |
| --- | --- | --- | --- | --- |
| # | Features | Score | Features | Score |
| 1 | NumDash | 0.3722 | SSLfinal_State | 0.715 |
| 2 | PctNullSelfRedirectHyperlinks | 0.3428 | URL_of_Anchor | 0.693 |
| 3 | PctExtNullSelfRedirectHyperlinksRT | 0.3064 | Prefix_Suffix | 0.349 |
| 4 | NumDots | 0.2941 | web_traffic | 0.346 |
| 5 | PctExtHyperlinks | 0.2597 | having_Sub_Domain | 0.298 |
| 6 | NumSensitiveWords | 0.2552 | Request_URL | 0.253 |
| 7 | PathLevel | 0.2295 | Links_in_tags | 0.248 |
| 8 | FrequentDomainNameMismatch | 0.1810 | SFH | 0.221 |
| 9 | ExtMetaScriptLinkRT | 0.1696 | Google_Index | 0.129 |
| 10 | HostnameLength | 0.1692 | age_of_domain | 0.121 |
| 11 | NumDashInHostname | 0.1504 | Page_Rank | 0.105 |
| 12 | NumQueryComponents | 0.1474 |  |  |
| 13 | SubmitInfoToEmail | 0.1126 |  |  |
