# Supplementary material for: Comparative evaluation of machine learning algorithms for phishing site detection
Source: PeerJ Comput Sci. 2024 Jun 24;10:e2131. doi: 10.7717/peerj-cs.2131 (PMC11232597; doi:10.7717/peerj-cs.2131)
Supplement: Table S7 [file peerj-cs-10-2131-s014.docx]

**Table S7.** The tuned hyperparameters for all models

| Model | Hyperparameters | Tuned Hyperparameters |
| --- | --- | --- |
| LR | penalty: [l1, l2, elasticnet] | penalty: l2 |
|  | C: [0.001, 0.01, 0.1, 1, 10, 100] | C: 0.1 |
|  | solver: [newton-cg, lbfgs, saga, liblinear] | solver: saga |
|  | max_iter: [100, 500, 1000] | max_iter: 500 |
| KNN | n_neighbors: [2, 3, 4] | n_neighbors: 3 |
|  | algorithm: [auto, ball_tree, kd_tree, brute] | algorithm: brute |
| DT | criterion: [gini, entropy] | criterion: Gini |
|  | max_depth: [2, 3] | max_depth: 3 |
|  | min_samples_leaf: [5, 6] | min_samples_leaf: 5 |
| RF | n_estimators: [50, 100, 150, 200] | n_estimators: 150 |
|  | max_depth: [None, 10, 20] | max_depth: 10 |
|  | min_samples_split: [2, 5, 10] | min_samples_split: 5 |
|  | min_samples_leaf: [1, 2, 4] | min_samples_leaf: 2 |
|  | max_features: [auto, sqrt, log2] | max_features: log2 |
| SVM | C: [0.5, 0.7, 0.9, 1] | C: 0.7 |
|  | kernel: [rbf, poly, sigmoid, linear] | kernel: sigmoid |
| XGBoost | learning_rate: [0.01, 0.1, 0.2] | learning_rate: 0.2 |
|  | n_estimators: [50, 100, 200] | n_estimators: 100 |
|  | max_depth: [3, 4, 5] | max_depth: 5 |
|  | min_child_weight: [1, 2, 3] | min_child_weight: 2 |
|  | subsample: [0.8, 1.0] | subsample: 0.8 |
|  | colsample_bytree: [0.8, 1.0] | colsample_bytree: 1.0 |
| CNN | number of filters: [32, 64, 128] | number of filters: 64 |
|  | filter size: [(3, 3), (5, 5)] | filter size: (3, 3) |
|  | pool size: [(2,2), (3, 3), (5, 5)] | pool size: (3, 3) |
|  | dense neurons: [128, 256, 512] | dense neurons: 128 |
|  | dropout rate: [0.25, 0.5, 0.75] | dropout rate: 0.5 |
| DL | Optimizer: [adam, sgd, rmsprop] | Optimizer: adam |
|  | Learning rate: [0.001, 0.01, 0.1] | Learning rate: 0.1 |
|  | Batch size: [16, 32, 64] | Batch size: 32 |
|  | dropout rate: [0.25, 0.5, 0.75] | dropout rate: 0.25 |
