# Supplementary material for: Comparative evaluation of machine learning algorithms for phishing site detection
Source: PeerJ Comput Sci. 2024 Jun 24;10:e2131. doi: 10.7717/peerj-cs.2131 (PMC11232597; doi:10.7717/peerj-cs.2131)
Supplement: Table S8 [file peerj-cs-10-2131-s015.docx]

**Table S8.** Models performance on both datasets before and after the hyperparameter tuning

| **Models** | **Accuracy** | **Precision** | **Recall** | **F1-score** | **FPR** | **Dataset** | **Before Hyperparameter Tuning (BHT)/After Hyperparameter Tuning (AHT)** |
| --- | --- | --- | --- | --- | --- | --- | --- |
| LR | 0.94 | 0.93 | 0.93 | 0.94 | 0.09 | 1 | BHT |
| KNN | 0.95 | 0.94 | 0.94 | 0.94 | 0.08 | 1 | BHT |
| DT | 0.97 | 0.97 | 0.96 | 0.96 | 0.09 | 1 | BHT |
| RF | 0.97 | 0.97 | 0.97 | 0.97 | 0.06 | 1 | BHT |
| SVM | 0.95 | 0.95 | 0.95 | 0.94 | 0.06 | 1 | BHT |
| XGBoost | 0.93 | 0.93 | 0.92 | 0.93 | 0.10 | 1 | BHT |
| CNN | 0.97 | 0.97 | 0.98 | 0.98 | 0.04 | 1 | BHT |
| DL | 0.95 | 0.95 | 0.94 | 0.96 | 0.06 | 1 | BHT |
| LR | 0.93 | 0.92 | 0.93 | 0.93 | 0.06 | 2 | BHT |
| KNN | 0.94 | 0.94 | 0.95 | 0.94 | 0.07 | 2 | BHT |
| DT | 0.96 | 0.96 | 0.96 | 0.96 | 0.06 | 2 | BHT |
| RF | 0.97 | 0.96 | 0.97 | 0.96 | 0.05 | 2 | BHT |
| SVM | 0.94 | 0.94 | 0.95 | 0.94 | 0.05 | 2 | BHT |
| XGBoost | 0.91 | 0.91 | 0.91 | 0.91 | 0.08 | 2 | BHT |
| CNN | 0.95 | 0.95 | 0.94 | 0.95 | 0.04 | 2 | BHT |
| DL | 0.94 | 0.95 | 0.93 | 0.94 | 0.07 | 2 | BHT |
| LR | 0.95 | 0.94 | 0.94 | 0.94 | 0.08 | 1 | AHT |
| KNN | 0.96 | 0.94 | 0.95 | 0.94 | 0.07 | 1 | AHT |
| DT | 0.97 | 0.97 | 0.97 | 0.97 | 0.06 | 1 | AHT |
| RF | 0.98 | 0.98 | 0.97 | 0.98 | 0.05 | 1 | AHT |
| SVM | 0.96 | 0.96 | 0.95 | 0.95 | 0.05 | 1 | AHT |
| XGBoost | 0.97 | 0.96 | 0.95 | 0.96 | 0.09 | 1 | AHT |
| CNN | 0.99 | 0.98 | 0.98 | 0.99 | 0.04 | 1 | AHT |
| DL | 0.98 | 0.98 | 0.97 | 0.97 | 0.05 | 1 | AHT |
| LR | 0.95 | 0.94 | 0.94 | 0.94 | 0.07 | 2 | AHT |
| KNN | 0.94 | 0.95 | 0.95 | 0.94 | 0.06 | 2 | AHT |
| DT | 0.97 | 0.97 | 0.96 | 0.96 | 0.05 | 2 | AHT |
| RF | 0.98 | 0.97 | 0.97 | 0.97 | 0.04 | 2 | AHT |
| SVM | 0.95 | 0.95 | 0.95 | 0.94 | 0.05 | 2 | AHT |
| XGBoost | 0.98 | 0.97 | 0.98 | 0.97 | 0.08 | 2 | AHT |
| CNN | 0.99 | 0.99 | 0.98 | 0.99 | 0.03 | 2 | AHT |
| DL | 0.99 | 0.98 | 0.98 | 0.98 | 0.04 | 2 | AHT |
